# Itinerário terapêutico de pessoas acometidas por amputação em decorrência de pé diabético: enredos de (des)cuidado

**DOI:** 10.1590/0102-311XPT187924

**Published:** 2025-11-01

**Authors:** Andressa de Andrade Santos, Adriano Maia dos Santos, José Andrade Louzado, Deise Lisboa Riquinho, Gabriela Garcia de Carvalho Laguna, Patty Fidelis de Almeida

**Affiliations:** 1 Universidade Federal da Bahia, Vitória da Conquista, Brasil.; 2 Escola de Enfermagem, Universidade Federal do Rio Grande do Sul, Porto Alegre, Brasil.; 3 Instituto de Saúde Coletiva, Universidade Federal Fluminense, Niterói, Brasil.

**Keywords:** Diabetes Mellitus, Itinerário Terapêutico, Acesso aos Serviços de Saúde, Atenção Primária à Saúde, Diabetes Mellitus, Therapeutic Itinerary, Access to Health Services, Primary Health Care, Diabetes Mellitus, Ruta Terapéutica, Acceso a los Servicios de Salud, Atención Primaria de Salud

## Abstract

O diabetes traz consigo implicações que exigem cuidado longitudinal e quando não controlado adequadamente é responsável por diversas complicações, dentre as quais o pé diabético. Este estudo, com abordagem qualitativa, analisa os itinerários terapêuticos de 20 indivíduos com amputação de membros inferiores em decorrência de pé diabético. A produção de dados envolveu entrevistas em profundidade e os dados foram interpretados mediante análise temática de conteúdo, com suporte teórico-metodológico da análise holística de itinerários terapêuticos. Foram eleitas duas categorias: (i) lidando com as vulnerabilidades - aspectos econômicos e sociais marcam as decisões no itinerário terapêutico; e (ii) percalços no caminho - a busca por assistência nas arenas de cuidado. Os sujeitos viviam em vulnerabilidade socioeconômica que se agravou com a amputação. A procura por cuidado na rede formal foi empreendida pelo próprio usuário. A ausência de orientação e encaminhamentos pela atenção primária à saúde e outros pontos de atenção podem ter retardado o cuidado oportuno. Paralelamente, foram utilizados cuidados informais, desde práticas religiosas até cuidados domésticos. O itinerário terapêutico das diferentes pessoas sinalizou a multiplicidade de experiências e o (des)cuidado que perpassa as distintas arenas.

## Abstract (en)

Diabetes has implications that require long-term care. When not adequately controlled, this disease can lead to several complications, including diabetic foot. The present qualitative study analyzes the therapeutic itineraries of 20 individuals with lower limb amputations as a result of diabetic foot. Data were collected through in-depth interviews and interpreted using thematic content analysis, with theoretical and methodological support from holistic therapeutic itinerary analysis. Two categories were chosen: (i) dealing with vulnerabilities - economic and social aspects influence the decisions made in the therapeutic itinerary; and (ii) mishaps along the way - the search for assistance in care settings. The individuals lived in situations of socioeconomic vulnerability that worsened with the amputation. Seeking care in formal health network was undertaken by the patients themselves. The lack of guidance and referrals from primary health care to other services may have led to delayed care. Informal care, ranging from religious practices to at-home care, was also used. The therapeutic itinerary of the different individuals revealed the multiplicity of experiences and care (or lack thereof) that permeates different settings.

## Abstract (es)

La diabetes conlleva implicaciones que exigen un cuidado longitudinal y, cuando no se controla adecuadamente, es responsable de diversas complicaciones, entre ellas el pie diabético. Este estudio, con abordaje cualitativo, analiza los itinerarios terapéuticos de 20 individuos con amputación de miembros inferiores a consecuencia de pie diabético. La producción de datos implicó entrevistas en profundidad y los datos fueron interpretados mediante análisis temático de contenido, con soporte teórico-metodológico del análisis holístico de itinerarios terapéuticos. Se eligieron dos categorías: (i) lidiando con las vulnerabilidades - los aspectos económicos y sociales marcan las decisiones en el itinerario terapéutico; (ii) contratiempos en el camino - la búsqueda de asistencia en las arenas de cuidado. Los sujetos vivían en vulnerabilidad socioeconómica que se agravó con la amputación. La búsqueda de cuidado en la red formal fue emprendida por el propio usuario. La ausencia de orientación y derivaciones por parte de la atención primaria de salud a otros puntos de atención puede haber retrasado el cuidado oportuno. Paralelamente, se utilizaron cuidados informales, desde prácticas religiosas a cuidados domésticos. El itinerario terapéutico de las diferentes personas señaló la multiplicidad de experiencias y el (des)cuidado que impregna las distintas arenas.

## Introdução

O itinerário terapêutico refere-se ao percurso que uma pessoa e sua família, com adoecimento ou não, fazem em busca por cuidado em saúde. Nesse caminho, traçam diferentes estratégias e recorrem a distintas modalidades de cuidado ^1^. Na busca pela resolução das necessidades de saúde, cada sujeito/coletivo traça um itinerário terapêutico multifacetado, que é resultante de diversas interfaces, o qual depende das possibilidades dos arranjos terapêuticos disponíveis (oficiais e não oficiais), mas, também, das racionalidades hegemônicas, da cultura comunitária, do modelo de proteção social do Estado, dos espectros religiosos, entre outros ^2^. 

Nesse mosaico, o aspecto socioeconômico apresenta-se como uma importante determinação social, pois pode figurar-se como um gerador de vulnerabilidades que acentuam as dificuldades de acesso ao cuidado oficial em sistemas de saúde ^3^. Além disso, o contexto das iniquidades sociais interfere nas escolhas e decisões sobre o cuidado ^4^ que, por sua vez, precisam ser analisadas numa perspectiva interseccional que considere gênero, raça/cor, etnia, idade, orientação sexual e identidade de gênero ^5^ ^,^ ^6^. Não obstante, os sujeitos/coletivos definem as suas escolhas, que variam desde a esfera institucionalizada da atenção à saúde - pública ou privada - até o campo “informal/não oficial” dos cuidados que perpassam a automedicação alopática, as práticas religiosas, os saberes e os curandeiros populares ^2^.

Outrossim, o adoecimento crônico como, por exemplo, o diagnóstico do diabetes, traz consigo uma condição de saúde que se perpetua por toda a vida e modifica atividades cotidianas, relações sociais, familiares e relações de trabalho ^7^. Há ainda, maior necessidade de acessar com frequência vários pontos da rede de atenção à saúde e criar vínculos com diferentes profissionais ^7^ ^,^ ^8^. O diabetes é uma doença crônica que, quando não controlada, pode desencadear diversas complicações, como insuficiência renal, complicações micro e macro vasculares, retinopatias e amputações ^9^. Embora muito grave, o pé diabético é uma das intercorrências mais comuns e responsável por maiores chances de amputação de membros inferiores ^10^, que acarretam em perda da capacidade de trabalho, mudanças na qualidade de vida da pessoa e sua família, impacto socioeconômico, além de reflexos emocionais e psicológicos que levam à sensação de incapacidade e dependência ^11^.

Grosso modo, o diabetes é uma condição sensível à atenção primária pois, por meio de políticas públicas efetivas e ações de cuidado desenvolvidas pelas equipes da Estratégia Saúde da Família (EqSF), é possível mitigar complicações, hospitalizações e óbitos decorrentes do descontrole da doença ^12^. Assim, o cuidado integral à pessoa com diabetes e com pé diabético é inerente à atenção primária à saúde (APS), uma vez que esse ponto da rede de atenção possui atributos essenciais para o desenvolvimento de estratégias que estimulem as práticas seguras de cuidado ^13^. A APS deve proporcionar suporte social à população com diabetes, por meio de intervenções sensíveis, centradas nas pessoas, e desenvolver uma compreensão sociocultural do adoecimento crônico ^14^.

Esse artigo analisa os itinerários terapêuticos de indivíduos de zonas rurais e urbanas em um município de médio porte, com amputação de membros inferiores em decorrência de complicações do diabetes mellitus caracterizadas como pé diabético.

## Aspectos metodológicos

Trata-se de um estudo com abordagem qualitativa ^15^ desenvolvido em um município do interior da Bahia, Brasil, com 370.879 habitantes ^16^, que possuía, em 2022, 13.501 pessoas com diabetes cadastradas na APS ^17^. Foram eleitas 20 pessoas adscritas em unidades de saúde da família (USF) da zona urbana e zona rural, a fim de diversificar as experiências do itinerário terapêutico. 

Utilizaram-se como critérios de inclusão: ter 18 anos ou mais, viver com diabetes e apresentar o evento traçador - amputação de membro inferior em decorrência de pé diabético. A identificação dos participantes foi realizada com o apoio da Diretoria de Atenção Básica do município, que forneceu os nomes dos usuários com a condição marcadora e as suas respectivas USF de adscrição. Foram contactados os enfermeiros e agentes comunitários de saúdes (ACS) das EqSF que intermediaram o contato com os usuários. A seleção dos sujeitos ocorreu por intencionalidade e o número de entrevistados foi definido pela reincidência de informações. Foram excluídas pessoas com amputações por outras causas, como, por exemplo, as amputações traumáticas.

A estratégia para a construção dos itinerários terapêuticos foi composta pela realização de entrevistas em profundidade com a utilização de roteiro semiestruturado. Essas foram agendadas previamente pelos ACS e ocorreram nos domicílios dos participantes. Todas as entrevistas foram realizadas pela mesma pesquisadora, com gravação de áudio (média de 40 minutos). A coleta de dados transcorreu no período de outubro a novembro de 2022.

**Figura 1:**
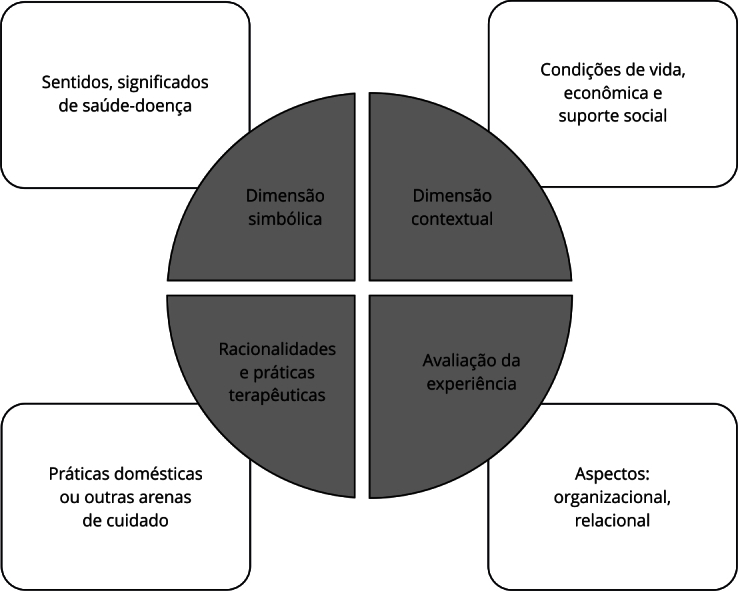
Modelo de análise holística de itinerários terapêuticos.

Os resultados emergiram de interpretações feitas por meio da análise temática de conteúdo ^18^, a partir do referencial metodológico da análise holística de itinerários terapêuticos ^19^. Para análise, as transcrições foram lidas exaustivamente e os núcleos temáticos (NT) selecionados, agrupados e categorizados a partir das dimensões pré-estabelecidas pelo referencial (Figura 1). Os dados foram tratados e construídas as sínteses narrativas dos itinerários terapêuticos e a análise das experiências dos usuários. Nesse estudo, foram selecionadas duas dimensões para discutir aspectos do itinerário terapêutico: (a) dimensão contextual e (b) dimensão de racionalidades e práticas terapêuticas.

Fonte: baseado em Trad ^19^.

A dimensão contextual refere-se a características específicas dos sujeitos investigados, como elementos que configuram diferentes capitais (econômico, social e cultural), cujo perfil influenciará no grau de informação sobre saúde-doença, no comportamento frente aos problemas, bem como na capacidade de aceder aos recursos disponíveis. Além disso, aborda a existência e peculiaridades de redes de apoio e reúne informações relativas à infraestrutura básica e, principalmente, à sociosanitária existente no território a ser investigado ^19^.

A dimensão de racionalidades e práticas terapêuticas traz como foco as lógicas subjacentes às diferentes arenas de cuidado e às práticas desenvolvidas, incluindo o autocuidado ou as práticas domésticas de atenção, explorando as interfaces, assim como os pontos de tensão entre as distintas arenas. Evidencia agentes de cura/cuidado, os preceitos etiológicos, nosológicos e terapêuticos ^19^.

### Aspectos éticos

O estudo foi aprovado pelo Comitê de Ética em Pesquisa do Instituto Multidisciplinar em Saúde, Universidade Federal da Bahia (parecer nº 5.445.446; CAAE: 57629422.0.0000.5556).

## Resultados

Os resultados foram agrupados em duas categorias temáticas: (i) lidando com as vulnerabilidades: aspectos econômicos e sociais marcam as decisões no itinerário terapêutico; e (ii) percalços no caminho: a busca por assistência nas arenas de cuidado. 

### Lidando com as vulnerabilidades: aspectos econômicos e sociais marcam as decisões no itinerário terapêutico

Os entrevistados evidenciaram como as condições de vida desfavoráveis e as dificuldades econômicas comumente retardavam o acesso aos serviços de saúde em tempo oportuno. Entre os participantes, 17 eram aposentados e a renda mensal por domicílio variava entre um e dois salários mínimos. Um dos usuários aposentados relatou empréstimos consignados para custear gastos com o tratamento, endividamento que comprometeu a renda fixa da família, agravando a situação socioeconômica e, contraditoriamente, limitando o cuidado à saúde para outras comorbidades.

Dois entrevistados não possuíam renda fixa, sendo o Bolsa Família a única fonte de renda. Em alguns casos, esse auxílio era pago a outros membros da família, contribuindo com o orçamento familiar. Três usuários aguardavam as perícias médicas do Instituto Nacional do Seguro Social INSS (INSS) após a amputação e, entre esses, apenas um recebia auxílio-doença (NT-1).

As localidades visitadas na zona urbana, especialmente nas áreas periféricas, apresentavam residências com dificuldades de acesso geográfico às USF, situadas em territórios considerados de risco à violência, com vias públicas sem pavimentação, insuficiência de transporte público e de limpeza urbana. Todos os usuários residiam em casas de alvenaria, embora fossem residências muito simples. Na zona rural, os domicílios visitados estavam distantes das USF.

A condição socioeconômica foi agravada por conta da amputação, tendo em vista a necessidade de suspensão das atividades laborais, aumento nos gastos com medicamentos e deslocamentos, gerando impactos financeiros no entorno familiar. Destacou-se a solidariedade familiar para custear tratamentos necessários até que fossem concedidos os benefícios previdenciários. Constatou-se de modo recorrente, a ausência dos serviços públicos de saúde nos itinerários terapêuticos, fenômeno amplamente naturalizado pela população e que impulsionava a busca por alternativa no setor privado, agravando a vulnerabilidade financeira das famílias (NT-2).

Destacaram-se os gastos ampliados com o pagamento por desembolso direto para obterem consultas com especialistas na rede privada. O uso de clínicas privadas “populares” foi uma alternativa utilizada pelos usuários com intenção de acessar mais rapidamente tratamentos e exames especializados. Entretanto, a necessidade de pagamento para realização de consultas e exames certamente limitou a procura pelos serviços, retardando o acesso oportuno à esfera formal de saúde. Ademais, diante da necessidade de realização de exames e procedimentos de alto custo, a continuidade do tratamento na rede privada ficava inviável, condicionando retornos esporádicos e pontuais aos serviços públicos (NT-3).

O desembolso direto com especialistas, transporte, medicações e alimentação foram os principais responsáveis por comprometer os gastos familiares, sobretudo, aos residentes na zona rural. Nesse aspecto, a ausência de transporte sanitário foi um grande entrave ao acesso oportuno. As longas distâncias entre as residências e os serviços de saúde demandavam o pagamento de altos valores a transportes particulares (NT-4) ou contavam com o “apoio” de agentes públicos da própria comunidade, que ofereciam o transporte por um valor relativamente menor numa relação de favorecimento (NT-5). Toda essa problemática contribuiu para que uma amputação evitável se tornasse o desfecho entre os entrevistados e, na vivência com a amputação, gerasse às pessoas maiores dificuldades para deslocamento. 

Mesmo na zona urbana, as dificuldades com transporte e locomoção estavam presentes. A procura pelos serviços de saúde, sobretudo das USF, ocorria com mais regularidade antes da amputação. A perda do membro dificultou o uso do transporte público, com necessidade de outros meios como táxi e carros por aplicativos, contribuindo para o adiamento ou a diminuição das visitas aos estabelecimentos de saúde. Associada a isso, a infraestrutura precária, na zona rural e urbana, das vias públicas limitou a locomoção - especialmente daqueles que faziam uso de muletas ou cadeiras de rodas (NT-6).

Em síntese, os longos períodos de espera na rede pública, a fragmentação assistencial, as barreiras geográficas e econômicas que dificultaram o acesso à saúde em tempo oportuno contribuíram para o desfecho da amputação (NT-7). Tais dificuldades geravam insatisfação e descredito com o Sistema Único de Saúde (SUS), bem como abriam espaço para busca de acesso mediante clientelismo. Nesta perspectiva, as decisões e escolhas que caracterizavam os itinerários terapêuticos eram condicionadas por circunstâncias/contingências sanitárias e não, exatamente, a partir das necessidades de cuidado (Quadro 1). 

**Quadro 1:** Lidando com as vulnerabilidades: aspectos econômicos e sociais marcam as decisões no itinerário terapêutico.

TEMA	CÓDIGO	FALAS
Situação econômica	NT-1	“*Eu trabalhava como motorista de caminhão. Trabalhei 32 anos em empresa, de carteira assinada, tudo. E hoje, não posso rodar mais, trabalhar mais. E é difícil, porque a gente não tem uma renda fixa.* [Minha esposa] *recebe esse auxílio do governo e toma conta dos meninos da* [vizinha]*. Ela pegava uns doces pra vender também, mas chegava o dia de pagar, o povo não pagava. Aí mandei parar de vender. Mas, sexta-feira, já vou fazer a perícia*” (E9 - homem, 52 anos, zona rural) “*Quando não tenho dinheiro tomo emprestado e pago. Tomo do meu genro, tomo do povo, quando não tenho, porque o dinheiro é pouco. Por causa de empréstimo* [consignado] *esse mês mesmo, meu dinheiro veio 400 reais. O que é que a gente faz com 400 reais, pra água, luz, mercado? Não dá pra nada não. E meu marido é cheio das contas também, empréstimo. Agora mesmo, minhas meninas querem consertar esse chão* [piso da casa]*, mas como é que conserta. Já falei pra elas, vai deixando aí do jeito que tá*” (E17 - mulher, 77 anos, zona rural)
O adoecimento agrava a condição socioeconômica	NT-2	“*E sinto muita falta, porque eu trabalhava, eu era cozinheira de churrascaria. Depois que amputei, parei de vez, porque não tinha mais como. No começo foi bem difícil, mas aí foi todo mundo ajudando, porque aqui mora muita gente. Aí meu irmão ajudava, minha sobrinha, mas foi difícil. Até que graças a Deus, consegui o benefício*” (E7 - mulher, 64 anos, zona urbana)
Desembolso direto	NT-3	“*Às vezes, quando posso, eu pago particular também e faço. Eu tenho cartão da Amor Saúde*” (E20 - mulher, 70 anos, zona urbana) “*Aí depois o olho já começou a doer e fui lá, e ele* [oftalmologista] *disse que tinha que fazer umas aplicação. Nesse tempo, essas aplicação foi 4.500 reais. Meu marido pagou mil e quinhentos, minha filha pagou mil e quinhentos e meu filho pagou mil e quinhentos*” (E17 - mulher, 77 anos, zona rural)
Ausência de transporte sanitário dificulta o acesso ao serviço de saúde	NT-4	“*Agora, estou passando com um médico da perna, lá em* [Vitória da] *Conquista. Um exame, só com ele, é 220 reais, fora o carro que a gente paga pra ir pra lá pra Conquista. Aí, as vans vão, mas não vou nas vans não, porque elas ficam lá embaixo no Ceasa* [centro da cidade]*, e pra ir lá pra cima no hospital, não aguento ir a pé. Aí, tem que ir no carro, tem que procurar um carro aqui. A gente paga pra ir. Disse que agora já tá por 150 reais. Aí é assim, quando a gente tem dinheiro e pode pagar um carro, pronto, a gente vai. Mas quando não tem, fica aí. Porque além de pagar o carro, pagar consulta, fica muito caro. E ainda tem esses remédios tudo da perna que tomo, que é comprado*” (E12 - mulher, 68 anos, zona rural) “*Pra ir pra esses lugares* [serviços de saúde]*, ia nessas vans ou de ônibus. Mas, uso mais a van que é mais rápido, o ônibus faz um percurso muito grande. Pra o posto, vou de bicicleta que também é longe. O que era mais difícil era ter que descer no centro pra ir lá pra o São Vicente* [hospital]*. De pé, subindo aquela ladeira de moleta, depois descia, na hora assim de meio dia. Condições pra pagar, tinha hora que só tava com o dinheiro da passagem. Aí eu falava, não posso mexer, nem um cafezinho tem hora que não podia nem tomar. Acho que isso era o mais difícil. Tinha uns* [motoristas das vans] *que ainda passava e me deixava lá de frente, mas tinha outros que não*” (E8 - homem, 61 anos, zona rural)
Clientelismo	NT-5	“*Nisso tudo, quem me ajudou muito também foi um vereador que já foi meu pastor. Ele tem um carro que leva gente pra Conquista. Aí, pago 50 reais pra ir por semana, e vou duas vezes por semana, porque se for pagar um carro particular, vou pagar 150 reais, as dez fisioterapias vão dar 1.500 reais e não tem esse dinheiro. Não preciso de um remédio, de outra coisa? Então, não tinha como. E se fosse no meu carro ia ser a mesma coisa, porque tinha que pagar o motorista 100 reais e abastecer o carro. Aí vai outras pessoas nesse carro, aí a gente divide*” (E11 - homem, 49 anos, zona rural) “*Ainda bem que aqui tem umas vizinhas muito boas, ficou mais eu, ajudou muito. Só Deus pra pagar. Aí, meu marido me acompanhava, quando ele não podia, ia uma mulher aqui que também trabalhava na área de saúde. Ela trabalhava na política. Aí, por exemplo, se o carro fosse 40 reais, ela dava vinte. Aí tinha dia que ia muita gente, aí você dava uma vez, de outra vez já não dava*” (E10 - mulher, 58 anos, zona rural)
Barreiras geográficas reduzem a busca por assistência à saúde	NT-6	“*Ia muito no posto, mas depois que cortou a perna ficou muito ruim pra ir pegar ônibus. Nessas ruas sem calçar é muito difícil pra andar com essa cadeira. Quando chove pra você ver aí como fica, aí quase não vou mais no posto. Ia direto, porque tinha pressão alta e o diabetes*” (E15 - homem, 67 anos, zona urbana) “*Antes de perder a perna, às vezes, ia andando, às vezes, ia de ônibus, às vezes de Uber. E agora, só taxi. Geralmente por semana, sabe quantos taxi, pego? Nove. E não é Uber, é taxi. Porque minha cadeira de rodas ela é maior, e pelo fato dela ser maior o Uber não cabe. E não é nem que não cabe, eles, muitas vezes, não têm a boa vontade de saber se dar. E o taxi não, já aviso e eles mandam um sedan, que aí dar pra eu fechar a cadeira e ir. Esses dias precisei, chamei, chamei e nada de táxi, aí fui e chamei um Uber. Aí não fui com a cadeira, fui de andador. O andador é bom. Mas a cadeira é que realmente é pra eu me deslocar de casa. Porque, não sei o local que vou encontrar, se tem rampas, se tem escada, o que tem*” (E16 - mulher, 59 anos, zona urbana)
Longa espera para consultas e exames	NT-7	“*Depois paguei pra ir no* [angiologista]*, mas ele passou uns exames, mas falou que eu não ia ter condições de gastar. Aí,* [minha esposa] *levou esse relatório lá no posto e a médica pediu esses exames, esses aí que tá demorando* [exames que estão na central de regulação para marcação] [e]*, porque ninguém tem condição de fazer exame se não for lá pela prefeitura ou aqui pelo posto de saúde, aí a gente vai ficar a vida toda assim, sem fazer a angioplastia. Então isso aí* [amputação] *ainda acontece também por causa do poder público*” (E1 - homem, 64 anos, zona urbana). “*Quando é baratinho, igual um exame de laboratório, é quarenta conto* [reais]*, aí a gente paga pra fazer mais ligeiro. Agora tem uns exames que demoram. Botei uma ressonância já tem um ano. Não pude fazer pago, porque é caro. Agora que o pastor vai ver com uma moça que trabalha lá se ela consegue apressar um pouquinho, porque é mil e tantos reais uma ressonância*” (E18 - mulher, 68 anos, zona rural)

### Percalços no caminho: a busca por assistência nas arenas de cuidado

As pessoas traçavam diversos caminhos na busca por resolver suas aflições em saúde. Nesse percurso, acionavam as arenas formais da rede de assistência à saúde, bem como, modos de cuidar que ultrapassavam a esfera institucionalizada, como terapias domésticas e religiosidade. Ficaram evidentes que os caminhos do cuidado foram iniciados na APS, onde o diagnóstico da doença crônica foi realizado (NT-8). Os usuários relataram que, ao perceberem sintomas de adoecimento, a USF foi o primeiro ponto procurado para atenção à saúde. 

**Figura 2:**
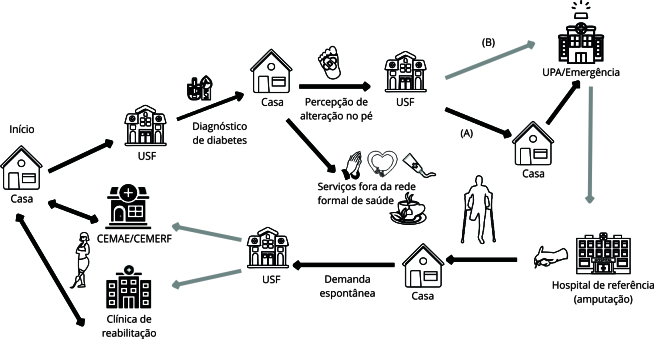
Diagrama dos itinerários terapêuticos de pessoas com pé diabético que buscaram a unidade de saúde da família (USF).

CEMAE: Centro Municipal de Atenção Especializada; CEMERF: Centro Municipal Especializado em Reabilitação Física e Auditiva; UPA: unidade de pronto atendimento. Fonte: elaboração própria. Nota: (A) não foram encaminhadas para outros serviços; (B) foram orientadas a procurar outro serviço; setas pretas: percursos realizados espontaneamente pelo sujeito; setas cinzas: percurso encaminhado por serviços de saúde.

Contudo, diante da complicação da doença crônica - o pé diabético -, outros pontos da rede de atenção à saúde foram acionados. Essa procura ocorreu, frequentemente, por iniciativa do próprio usuário. Apenas três entrevistados relataram que, após a busca por assistência na USF, foram orientados por profissionais de saúde a procurarem outros serviços mais adequados tecnicamente para a continuidade do cuidado, uma vez que a maioria dos usuários apresentava complicação avançada da doença (NT-9) (Figura 2).

A ausência de orientação e encaminhamentos por parte de profissionais de saúde a outros pontos de atenção pode ter sido um fator que retardou o cuidado adequado, desencadeando o desfecho da amputação (NT-10) (Figura 2).

**Figura 3:**
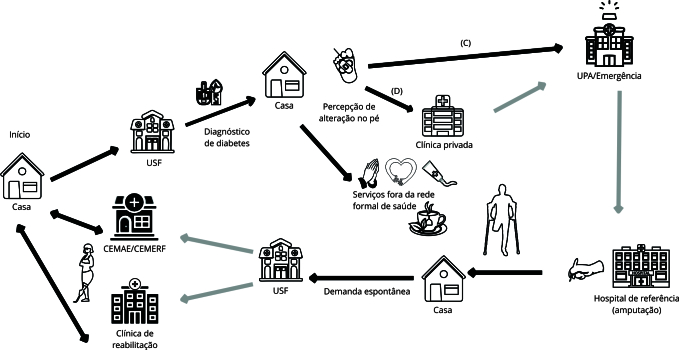
Diagrama dos itinerários terapêuticos de pessoas com pé diabético.

CEMAE: Centro Municipal de Atenção Especializada; CEMERF: Centro Municipal Especializado em Reabilitação Física e Auditiva; UPA: unidade de pronto atendimento; USF: unidade de saúde da família. Fonte: elaboração própria. Nota: (C) procuraram diretamente serviços de emergência; (D) procuraram diretamente o setor privado; setas pretas: percursos realizados espontaneamente pelo sujeito; setas cinzas: percurso encaminhado por serviços de saúde.

A maioria dos usuários procurou diretamente os serviços de emergência (NT-11), por entenderem que apenas esses serviços tinham capacidade de resolução. A unidade de pronto atendimento (UPA) e o hospital de referência de alta complexidade do município configuraram-se como os principais serviços demandados pela população. Nesse contexto, as narrativas revelaram percepções de pouca resolutividade da APS e evidenciou a valorização popular do modelo biomédico nas práticas de cuidado (Figura 3). 

A UPA foi, comumente, a porta de entrada para os serviços especializados e hospitalares. O primeiro acesso à rede de atenção via UPA, ainda que comum entre os entrevistados, era problemática e agravava o quadro clínico, pois havia longos períodos de espera para avaliação médica e para transferência para um hospital de referência. 

Entre os entrevistados, cinco, ao apresentarem os sintomas do pé diabético, decidiram buscar um especialista em serviços privados, em decorrência da lentidão da resposta no SUS (NT-12) (Figura 3). Entretanto, salienta-se que, em alguns casos, essa escolha deu-se independentemente da tentativa de busca pelo SUS, muitas vezes, motivada pela expectativa do não acesso e sobrevalorização dos serviços privados de saúde.

Entretanto, todos os casos que optaram por buscar o setor privado na produção de seus itinerários terapêuticos, posteriormente, necessitaram lançar mão da assistência no SUS, ora por não disporem de condições financeiras para arcar com a continuidade do tratamento, ora porque o setor privado não possuía estrutura técnica adequada para resolução do problema. 

Observou-se ainda que, após o período de internamento, os usuários retomaram o contato com a USF, geralmente pela necessidade de realização de curativos após a amputação e para futuros encaminhamentos a especialidades médicas. As avaliações com especialistas aconteceram no Centro Municipal de Atenção Especializada (CEMAE), que é um serviço de gestão municipal que dispõe de diversas especialidades médicas. Geralmente os encaminhamentos a esse serviço ocorreram por solicitações feitas pelo médico da USF (NT-13) (Figura 3).

Os resultados mostram que alguns usuários já se encontravam na fase de adaptação e aquisição de uma prótese. Para isso, acessavam o Centro Municipal Especializado em Reabilitação Física e Auditiva (CEMERF) que, também, era gerido pelo município (NT-13) (Figura 3). 

Outro serviço de saúde muito presente nos itinerários terapêuticos foi a Clínica Municipal de Reabilitação, especializada em tratamentos de feridas crônicas ou de difícil cicatrização que, também, fornecia atendimentos em fisioterapia e outras especialidades. O atendimento nesse serviço era “porta aberta”, sem necessidade de regulação, o que facilitava o acesso espontâneo. Os usuários geralmente procuravam esse serviço a partir da indicação de um profissional de saúde (NT-13).

Depois de solucionadas as demandas agudas da complicação da doença, geralmente, a procura pelos serviços de saúde, especialmente a USF, passou a ocorrer em momentos pontuais. Frequentemente, a USF foi procurada para renovação de receitas, retirada de medicamentos e realização de curativos. Notou-se que os principais vínculos entre usuários e profissionais ocorreram com os técnicos de enfermagem responsáveis pelo curativo, o ACS e o médico. As demais categorias profissionais tiveram menor representatividade nas narrativas (NT-14). 

Os itinerários terapêuticos mostraram que tanto os usuários da zona urbana quanto da rural traçaram caminhos semelhantes na esfera formal dos serviços de saúde, embora as dificuldades de acesso nos diferentes contextos tenham se apresentado com modo e intensidade distintos.

Concomitantemente às práticas terapêuticas do âmbito formal de saúde, os sujeitos buscavam outros modos de cuidar de si diante do adoecimento, a exemplo do autocuidado, automedicação, uso de medicamentos caseiros e religiosidade (Figuras 2 e 3). Havia, assim, um esforço para a manutenção do autocuidado. Embora muitas narrativas expressassem dificuldades de convivência com a doença crônica e suas limitações - especialmente com hábitos alimentares -, frequentemente os usuários buscavam moldar seus comportamentos cotidianos na tentativa de controlar a doença.

O autocuidado foi considerado uma relevante prática que contribuía na redução da reincidência de complicações da doença, embora a coparticipação no cuidado só tenha sido percebida como importante frente ao agravamento dos quadros (NT-15).

A automedicação foi uma prática comumente utilizada. O uso de medicamentos alopáticos sem prescrição médica ocorreu com intuito de resolver o problema de saúde sem a necessidade de procurar por um serviço da rede oficial. Essa escolha feita pelos usuários pode ter ocorrido diante da dificuldade de acesso aos serviços de saúde. No entanto, a expectativa de melhora e/ou cura da doença com a automedicação adiou a busca por cuidado na arena institucional (NT-16).

O saber popular foi valorizado nas narrativas. Os cuidados domésticos, o uso de chás e unguentos revelaram o reconhecimento acerca da importância das práticas de cuidado domésticas para recuperação da saúde (NT-17). Não obstante, apesar da relevância, ficou evidente que a maioria das pessoas as utilizavam como terapia complementar à medicina convencional. Os cuidados domésticos, na maior parte dos casos, estavam associados ao uso de medicações alopáticas prescritas por um médico.

Dois usuários referiram não fazer uso de nenhuma terapia doméstica priorizando o cuidado biomédico. Entretanto, o saber biomédico também foi relativizado diante do desfecho desfavorável. 

A prática religiosa mostrou-se presente nos itinerários terapêuticos, pois proporcionou esperança e perseverança no enfrentamento dos problemas de saúde. A vivência do adoecimento despertava fraquezas e medos que podiam favorecer a descontinuidade da assistência. Nesse contexto, a religiosidade teve relevante papel no processo de enfrentamento da doença - a fé e a crença em milagres -, pois devolveu a expectativa de cura, propiciando resiliência (NT-18).

A comunidade religiosa foi uma importante rede de apoio. Os vínculos estabelecidos com outras pessoas por meio da religião deram suporte espiritual no enfrentamento da doença. Essa conexão com a religião, em alguns casos, já era parte cotidiana da vida das pessoas, e em outros, foi desenvolvida após o adoecimento (NT-18). Durante as visitas para realização das entrevistas foram observados objetos religiosos nas casas e uso de acessórios religiosos (Quadro 2).

**Quadro 2:** Percalços no caminho: a busca por assistência nas arenas de cuidado.

TEMA	CÓDIGO	FALAS
Diagnóstico do diabetes na APS	NT-8	“*Tem muito tempo que tenho diabetes, fui lá no posto, aí foi lá que descobri. O médico mediu e viu que tava com diabetes, mas já tem tempo. Lembro que sentia assim que fazia xixi direto, tinha vez que perdia o sono e comia doce*” (E14 - mulher, 71 anos, zona urbana)
Pé diabético e encaminhamento na APS	NT-9	“*Fui no posto, chegou a médica falou pra mim: ‘oh seu pé não tem jeito aqui não. Você vai ter que ir no Hospital de Base, que lá que mexe com essas coisas’. Aí, primeiro fui pra UPA, fiquei uns quatro dias, porque não tinha vaga, fiquei ali naquela salinha dormindo numa cadeira. Aí, depois surgiu a vaga, me mandaram pra cá pro Hospital de Base, chegou lá, me internaram logo*” (E18 - mulher, 68 anos, zona rural)
Ausência de encaminhamento na APS	NT-10	“*Quando começou a mancha, fui no posto. Mas ninguém me mandou procurar outro lugar não. Eu mesmo fui vendo se melhorava.* [Minha esposa] *me levou pra o hospital. Eu mesmo não tava querendo ir não, mas ela falou: ‘Vamos lá pra ver’. Aí, no começo da semana tinha ido no posto. Quando foi no domingo, aumentou rápido. Começou a empretecer o pé, aí falei, é, não tem jeito não, vamos lá*” (E9 - homem, 52 anos, zona rural)
Busca por serviços de emergência	NT-11	“*Quando fiquei internada foi lá no Hospital de Base. Fui direto pro hospital, porque sempre que a gente sente assim esses problemas o povo fala que é lá que resolve. Aí, já fui direto pra lá*” (E12 - mulher, 68 anos, zona rural) “*Fiquei uns 15 dias internado pra ver se o sangue circulava e o sangue não circulou. Aí, fiz outros exames e teve que amputar a perna. Mas fiquei uns 10 dias na UPA, até surgir a vaga da cirurgia no Hospital de Base. Fiquei uns 30 a 40 dias internado entre a UPA e o Hospital de Base*” (E3 - homem, 74 anos, zona urbana)
Busca por serviços de saúde privado	NT-12	“*Aí, fui peguei daqueles táxis* (...)*, falei pra o moço, oh você não me leva nem pra UPA, nem pra o Hospital de Base, me leva pro Unimec* [hospital privado]*. Aí, na segunda, fui no médico, no meu cardiologista. Paguei a consulta, mas como era bem no tempo da pandemia, ele falou que não podia resolver nada. Ele falou: ‘você vai ou pra UPA ou pro Hospital de Base’. Aí, minha sobrinha que é nojenta falou: ‘lá você não vai não. Vumbora pro Unimec* [hospital privado]*’. Chegou lá, me botou o dia todo de castigo lá. Esse médico é particular, era 300 reais. Aí, a gente pagou logo. Esse médico fez exame de sangue, ultrassom, fez tudo. Quando foi 5 horas da tarde, ele chegou na cara de* [minha sobrinha] *e falou: ‘não, esse caso, não resolvemos não. Esse caso, é caso de amputação, você tem que levar ou pra UPA ou pra o Hospital de Base’*” (E11 - homem, 49 anos, zona rural)
Acesso aos serviços de saúde após amputação	NT-13	“*Aí, ele* [cirurgião] *passou os remédios e falou: ‘você precisa só tomar os remédios’. Ele deu os relatórios, aí, quando preciso, vou no posto e o doutor* [médico da EqSF] *faz os encaminhamentos. Ele que mandou ir no angiologista, ele que me deu as guias, faz esse trabalho todo*” (E3 - homem, 74 anos, zona urbana) “*Depois de lá do hospital, eles* [médicos] *mandou ir lá pra o CEMAE. Sei que fui lá um bocado de vezes, fazer exame com o médico lá do CEMAE, pra ele me olhar e tal.* (...) *E fui no CEMERF tirar as medidas pra fazer a prótese, isso foi quase um ano depois da cirurgia* (...) *Tenho só que acostumar com a prótese*” (E9 - homem, 52 anos, zona rural) “(...) *Aí, o rapaz concordou, mas falou: ‘é, mas vou encaminhar a senhora pra clínica de reabilitação, porque lá tem os profissionais de fazer o tratamento, lá os materiais não são comuns’. Porque geralmente quando a gente vai no posto é mais de primeira urgência, é gaze. E lá não, é espuma, tem placa hiper cara, tratamento adequado. E ia fazia os curativos lá na clínica.* (...) *A clínica lá foi excelente. Ainda tô lá, só que agora numa nova versão, na fisioterapia, porque vou colocar a prótese e tem que fazer* (E16 - mulher, 59 anos, zona urbana)
Busca pela ESF	NT-14	“*No posto, eu demorava de ir, mas ia. E depois que começou essa perna que comecei ir na UPA. No posto, quem mais me atende é o doutor* [e] *vou no posto mais por causa da pressão e do diabetes e é só ele* [médico] *e a menina do curativo, que me atende*” (E3 - homem, 74 anos, zona urbana) “*No posto, costumo ir. O remédio da pressão, pego no posto. E a insulina, também, pego no posto. E quem mais me atende lá é* [a ACS]*. Aí sempre quando preciso assim, que adoeço sempre vou no posto. Mas já tem tempo que não vou*” (E12 - mulher, 68 anos, zona rural)
Autocuidado	NT-15	“*Agora, parei de comer muito doce, refrigerante, porque eu era doida por causa de refrigerante. Meu café, tô bebendo amargando, porque tem o adoçante, mas não dou muito bem. Parei mais com negócio de massa. Pão mesmo, tirei mais. E esses dias, passei até aqui na nutricionista do posto. Ela passou um monte de coisa pra mim comer. Arroz, macarrão, pão, tudo integral. Pão mesmo, só como uma vez no dia, porque comia muito pão. Esse negócio de massa, beiju, essas coisas tudo tirei mais. Aí, vai controlando*” (E10 - mulher, 58 anos, zona rural) “*Comprei a faixa, adaptei uns aparelhos com garrafa pet, areia, uso o próprio andador, pra não parar. Porque o restante da perna tem que ficar bem flexível, ele não pode ficar duro, enrijecido. Minha parte, eu to fazendo. Porque o possível a gente faz, e o impossível a gente entrega pra Deus. Então, to fazendo, é tá vigiando com o que come, tá fazendo a fisioterapia. As coisas que o médico orienta, to fazendo* (E16 - mulher, 59 anos, zona urbana)
Automedicação	NT-16	“*Aí é difícil o atendimento,* [e] *aí às vezes aqui em casa mesmo se ela [taxa glicêmica] tiver muita alta, eu mesmo aumento a dose da insulina pra ver se consigo baixar*” (E6, homem, 53 anos, zona urbana) “*No pé, eu passava umas coisas, o pessoal falava ‘ah isso aqui é bom’, aí continuei, mas acho que foi pior pra mim. Nem lembro o que usava, porque um e outro me davam um remédio pra ir passando, aí naquela ansiedade pra sarar, ia passava uma pomada, passava o que mandava e por cima tava melhorando, mas por dentro ela* [lesão no pé] *tava viva* [sem apresentar melhora]*. Achei que tava melhorando, mas não tava. Cheguei a essa conclusão*” (E8 - homem, 61 anos, zona rural)
Cuidados domésticos	NT-17	“*Ele* [o pé] *começou a enroxar a unha, aí depois foi crescendo, crescendo, fui esperando, colocando remédio pra ver se sarava, usava remédio do mato. Muita gente já me deu explicação de um remédio que é bom, sempre as pessoas me ensinou. Uma coisa que dou bem mesmo quando ela* [taxa glicêmica] *tá bem alta é folha de graviola, faz o chá. Se tiver de 500* [taxa glicêmica] *e você tomar em 40 minutos baixa* (E6 - homem, 53 anos, zona urbana) “*Aí, chegou aqui minha menina começou cuidar. Ela pegava babosa, mastruz, fazia aquela papa, passava, enrolava e eu tomava os medicamentos, os antibióticos. Aí foi cuidando, cuidando com tanto amor, pois tem hora que amor resolve problema, pois meu pé sarou.* (...) *E tomo muito chá, as folhas da árvore servem de remédio* [e] *meço minha diabetes, tomo chá, ela fica boa* (E17 - mulher, 77 anos, zona rural)
Religiosidade	NT-18	“*Aí você pede, bate o joelho no chão que Deus ouve tudo. Mas, aí pra você ver, Deus faz tudo certo. Passei apurada, fiquei de cadeira de rodas, moleta, deitada, sentido dor e mais dor,* (...) *e Deus deu tudo pra mim. Cheguei minha idade, deu meu aposento. Por isso que falo, tem que ter muita fé, oração e tudo. Aí, sempre vou na igreja, peço oração, rezo o terço* [e] *graças a Deus, pedi muito a Deus, porque fiquei ruim mesmo. Só não morri, porque não foi o dia de Deus levar. Sou da igreja católica. Pedi muita oração. Esse povo aqui, os vizinhos, fez muita oração. É a fé, quem tem fé em Deus*” (E10 - mulher, 58 anos, zona rural) “*Procurei uma igreja de crente aqui mesmo e fui muito abençoada, porque só andava de bengala. Aí quando foi um dia o pastor me rezou e falou assim: ‘de hoje em diante, a senhora não vai mais usar bengala’. Acho que não sei, a fé. Porque, eu tava doida pra ter saúde mesmo. Aí desse tempo pra cá, não usei mais a bengala* (E12 - mulher, 68 anos, zona rural) “*Então, quando a gente começar a fazer essas indagações e a gente mesmo responder com teoria bíblica a coisa muda de figura. A dor, o peso, o sofrimento, tudo diminui*” (E16 - mulher, 59 anos, zona urbana)

APS: atenção primária à saúde; CEMAE: Centro Municipal de Atenção Especializada; CEMERF: Centro Municipal Especializado em Reabilitação Física e Auditiva; EqSF: equipes da Estratégia Saúde da Família; UPA: unidade de pronto atendimento.

## Discussão

Os resultados destacam que os itinerários terapêuticos se desenvolviam por caminhos entrecruzados. Determinantes sociais como baixa escolarização e renda, escassez de informação sobre os cuidados em saúde, restritas oportunidades de emprego e condição precária de moradia geram um contexto desfavorável às práticas de autocuidado e ao acesso oportuno a serviços de saúde ^3^ ^,^ ^20^. Existe forte relação entre desigualdades socioeconômicas e prevalência de doenças crônicas, especialmente entre indivíduos com raça/cor preta ou parda, não alfabetizados, com Ensino Fundamental incompleto, que não possuem plano de saúde privado e com menor renda ^21^.

Pessoas que residem em localidades com forte desigualdade social estão mais vulnerabilizadas e têm menores chances de, ao desenvolverem diabetes, acessarem os cuidados requeridos de forma adequada, contínua e em tempo oportuno ^22^, como observado neste estudo. No mesmo sentido, pessoas em piores condições sociais têm menos oportunidades e possibilidades de escolhas, requerendo que o Estado assuma a responsabilidade por garantir direitos sociais amplos, particularmente para mitigar barreiras de acesso à saúde ^23^.

A condição financeira desfavorável dificulta o acesso a diferentes serviços e políticas públicas, revelando iniquidades sociais que repercutem sobre a saúde ^24^. Nessa direção, o Bolsa Família, por exemplo, mostrou-se uma importante ferramenta de assistência social às famílias mais vulnerabilizadas ^25^ ^,^ ^26^. 

A dimensão programática da vulnerabilidade se expressa na forma como os serviços de saúde respondem às necessidades das pessoas para a garantia de cuidado integral ^27^. O que se observou é que tanto as vulnerabilidades sociais quanto programáticas forçaram as pessoas a buscarem serviços de saúde somente quando o estado de saúde se agravava, retardando o cuidado adequado ^28^, incorrendo em complicações como, por exemplo, a amputação por pé diabético. 

O adoecimento, nesse estudo, agravou a situação financeira das famílias diante da necessidade de desembolso direto para assistência à saúde. O comprometimento da renda com pagamentos em cuidado à saúde leva os mais pobres a sofrerem com gastos catastróficos, exacerbando a situação de iniquidade ^29^. No mundo, estima-se que, em 2017, quase um bilhão de pessoas gastaram mais de 10% de seus orçamentos domésticos com assistência à saúde e, entre esses, 290 milhões gastaram mais de 25%, agravando o empobrecimento e a pobreza extrema ^30^. No Brasil, mesmo com o SUS, as famílias têm participado expressivamente dos pagamentos diretos em saúde ^31^. Em 2019, os gastos em saúde corresponderam a 9,6% do produto interno bruto (PIB), sendo que 5,8% foram relativos à participação das famílias e 3,8% do governo, evidenciando um percentual de participação familiar superior aos gastos públicos ^32^.

As dificuldades de acesso foram mais expressivas na esfera da assistência especializada, com a quebra da continuidade e busca de serviços privados ^33^. A problemática de acesso é mais expressiva nas localidades rurais e remotas ^34^. As barreiras geográficas, como as distâncias das localidades rurais aos serviços especializados e hospitalares, as más condições das estradas de acesso no meio rural, a ausência de transporte sanitário ou a presença insuficiente em dias e horários restritos, dificultam o acesso dessa população aos serviços de saúde, gerando interrupção frequente do cuidado longitudinal ^35^ ^,^ ^36^. Essa realidade destaca a necessidade de implantação de medidas, a exemplo do cofinanciamento para o transporte, a fim de que se reduzam as iniquidades de acesso à saúde das populações rurais ^33^.

Além das despesas com transporte, há ainda custos com consultas e, principalmente, com exames diagnósticos em decorrência da lentidão da rede pública em ofertar assistência especializada. Assim, o desembolso direto é utilizado como alternativa de acesso diante das lacunas assistenciais e da oferta insuficiente do SUS ^33^ ^,^ ^37^. Ademais, problemáticas do acesso estão associadas ao subfinanciamento, bem como a uma gestão deficiente do SUS ^38^. Os obstáculos de acesso levam os usuários à procura por clínicas populares que, frequentemente, não asseguram tratamento contínuo e levam essas pessoas a retornarem aos serviços de saúde público ^39^.

A escassez e as dificuldades de acesso estimulam a privatização do espaço público e a obtenção de assistência por meio de práticas clientelistas que privilegiam os interesses individuais, negam o direito universal à saúde e acirram as iniquidades ^40^. Tais práticas se fortalecem em contextos de maior desigualdade socioeconômica e ausência de proteção social do Estado, operando especialmente sobre as camadas populares ^41^ ^,^ ^42^. 

Todos estes determinantes incidiram sobre os itinerários terapêuticos, sendo os usuários majoritariamente responsáveis por criar seus próprios caminhos para alcançar os serviços de saúde. Para isso, os sujeitos desenvolvem fluxos que, muitas vezes, fogem dos trajetos formais, procurando a porta de entrada mais acessível, na expectativa de ter suas demandas atendidas. Por vezes, fazem consecutivas aproximações, “furando” filas e “descumprindo” regras, entrando nas portas que se mostram abertas e desenhando seus mapas terapêuticos de cuidados ^43^.

Os usuários pouco reconheceram a APS como o lugar para resolução de seus problemas de saúde, comumente vista como local de acesso para busca de procedimentos simples. As ações de promoção de saúde e prevenção das doenças crônicas são secundárias, levando os usuários a utilizarem diversas portas de entrada ^8^. Tais achados reforçam que a Estratégia Saúde da Família (ESF) precisa, portanto, promover em seu território estratégias de busca ativa de pessoas com doenças crônicas, a fim de identificar e conhecer os usuários adscritos, garantindo assistência adequada, cuidado longitudinal e cumprimento das ações de cuidado às condições sensíveis à APS, como o diabetes ^44^. 

A assistência hospitalar e os serviços de emergência foram acionados prioritariamente aos serviços da APS. Outrossim, a forte influência do modelo biomédico na sociedade e sobre os serviços de saúde contribui para a valorização do cuidado especializado, em detrimento do cuidado longitudinal na APS ^45^. De tal modo, estimulados a buscar por uma causa biológica do adoecimento, e a esperar no especialista e nos modernos exames um caminho para a cura ^46^, os sujeitos procuram por alguém que interprete seu adoecimento e lhes ofereça tratamento. Além disso, depositam sua confiança no profissional, nos exames, nos medicamentos, ampliando sua dependência à medicalização e reduzindo sua autonomia ^47^.

As fragilidades comunicacionais foram fatores limitantes para a continuidade dos cuidados. A inexistência de mecanismos que facilitem o acesso ágil à informação gerou desencontros nas condutas e dificultou o cuidado longitudinal. Assim, a limitada coordenação do cuidado tem como um dos entraves a insuficiente disponibilidade de tecnologias de informação ou a resistência em utilizá-las quando disponíveis, o que dificulta o diálogo interprofissional ^48^. 

Igualmente, a ausência de fluxos definidos nas redes de atenção à saúde reforça esse obstáculo ao não estabelecer mecanismos de contrarreferência, deixando a APS à mercê do retorno espontâneo dos usuários ou seus familiares, tornando-os os principais (ou únicos) interlocutores de informações terapêuticas. A ausência de registro informatizado dificulta sobremaneira o acompanhamento do plano terapêutico de cada sujeito, obstruindo a continuidade do cuidado ^49^.

Para além dos cuidados na rede institucionalizada, diante da experiência do adoecimento os sujeitos tiveram diferentes dimensões de sua existência mobilizados e lançaram mão de distintas formas de cuidado em seu itinerário terapêutico, quando buscaram de forma complementar outros modos de cuidar e fazer em saúde, com destaque aos cuidados influenciados pelo saber popular. Os sujeitos mobilizaram saberes na busca de melhor gerir o autocuidado, sempre levando em consideração o que acreditam ser o melhor para si ^50^.

A convivência com o adoecimento crônico expôs potencialidades culturais, históricas e intergeracionais como o uso de chá e sucos fitoterápicos como um recurso adjuvante ao tratamento alopático. Assim, diante da pluralidade social, as pessoas usam modos diferentes de enfrentar o adoecimento a partir de significados e símbolos que compõem o processo saúde-doença-cuidado ^51^. Igualmente, ao longo do processo de enfrentamento do adoecimento, os sujeitos emergem sob suas dimensões subjetivas, buscando dar novos sentidos e significados à experiência, na expectativa de trilhar caminhos de cuidado ^52^. 

Nesse contexto, a espiritualidade também foi evocada como suporte no enfrentamento da doença. A convivência com o ambiente religioso pode propiciar práticas de autocuidado visto que muitas religiões mantêm orientações morais e práticas quanto aos cuidados com a saúde ao considerarem o corpo humano como sagrado, estimulando, assim, seus integrantes a cuidarem melhor de si ^53^. Ao reunir pessoas com propósitos comuns, a religiosidade cria um forte sistema de apoio social capaz de auxiliar no enfrentamento do adoecimento com menos sofrimento, ou maior resiliência ^53^.

## Considerações finais

Os itinerários terapêuticos desvelaram a multiplicidade de experiências e o (des)cuidado que perpassa as distintas arenas, culminando no desfecho extremo da amputação. 

O estudo ratifica a necessidade de alternativas de agendamento que facilitem a consulta de retorno com especialista, para o alcance de melhor acompanhamento e eficácia no tratamento; cofinanciamento para o transporte e/ou a oferta desse, sobretudo nas localidades rurais; ampliação de recursos materiais, financeiros e parcerias que possibilitem a implantação de atividades práticas de educação em saúde, aumentando o conhecimento dos usuários para o autocuidado.

Por fim, sem excluir o cuidado biomédico, o processo de trabalho dos profissionais de saúde deve ir ao encontro de uma produção de atenção que valorize os aspectos simbólicos dos sujeitos, haja vista esse estudo tenha revelado que esses contribuem como estratégias de enfrentamento da doença.

## Data Availability

Os bancos de dados utilizados no estudo, incluindo os códigos de extração, análises e resultados estão disponíveis em repositório: Universidade Federal da Bahia https://repositorio.ufba.br/handle/ri/37843. The sources of information used in the study are indicated in the body of the article.
